# Recurrent Meningeal Hemangiopericytoma with Multiple Metastasis and Hypoglycemia: A Case Report

**DOI:** 10.1155/2012/628756

**Published:** 2012-09-16

**Authors:** Jammy Kin Iong Chan, Wah Cheuk, Luen Cheong Ho, Jian-Ming Wen

**Affiliations:** ^1^Department of Pathology, Kiang Wu Hospital, China; ^2^Department of Pathology, Queen Elizabeth Hospital, Hong Kong

## Abstract

*Aims*. We report on the unusual case of a 43-year-old man who developed recurrent meningeal hemangiopericytoma and presented with hypoglycemia 6 years after excision of the tumor. *Methods and Results*. We utilized computed tomography to assure multiple tumor metastasis and cranial recurrence of previous meningeal hemangiopericytoma and clinical laboratory tests and immunohistochemical staining to characterize this case. Magnetic resonance imaging and computed tomography showed the recurrent tumor at original torcular site was increased in size. Abnormal low levels of growth hormone, insulin, and insulin-like growth factor-I except insulin-like growth factor-II were detected in the serum. By immunohistochemistry, the neoplastic cells characteristically express diffusely CD99, bcl2, and variable CD34. After radio- and chemotherapy, serum glucose level of the patient returned to normal. *Conclusions*. Comparing other brain tumors, meningeal hemangiopericytoma has a higher recurrent and metastatic rate, but this tumor with hypoglycemia is very rare.

## 1. Introduction

Hemangiopericytoma is a mesenchymal tumor arising from pericytes. It was firstly reported by Stout and Murray [1] and occurs mostly in soft tissue, including brain meninges primarily. In the 2007 WHO Classification of Tumors of the Central Nervous System, meningeal hemangipericytoma is a rare neoplasm and constitutes about 0.4% of CNS tumors [2]. It is rather aggressive, easily recurrent, and often extracranially metastatic. We report here a case of meningeal hemangiopericytoma with extracranial metastasis, accompanying rarely hypoglycemia.

## 2. Case Report

**Figure 1:**
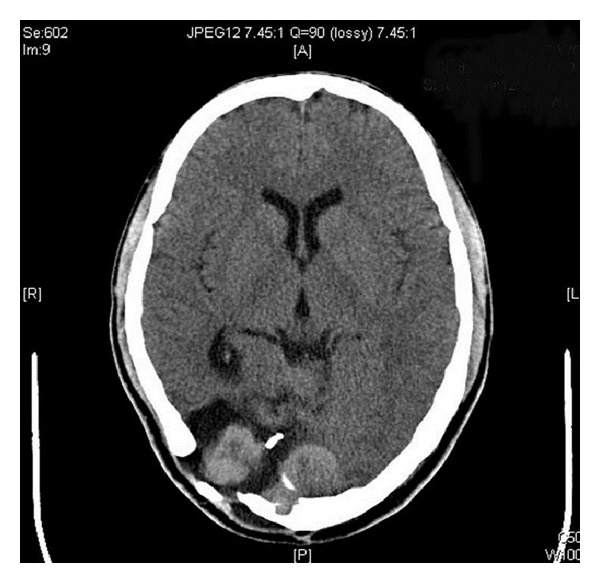
MRI scan demonstrates the recurred tumor mostly involved right posterior fossa.

A 43-year-old smoker man presented with left blurring of vision and tinnitus six years before. Cranial computed tomography (CT) demonstrated a torcular mass with changes of surgical excision and supplementary Gamma-knife therapy at the primary hospital. Meningeal hemangiopericytoma was diagnosed postsurgically. Six years later, a followingup computed tomography of the patient revealed a small recurrence of meningeal tumor and then treated with Gamma knife. Thereafter, magnetic resonance imaging (MRI) and CT (Figure 1) showed the recurrent tumor at original torcular site was increased in size. Craniotomy with total excision of the tumor was done. 

**Figure 2:**
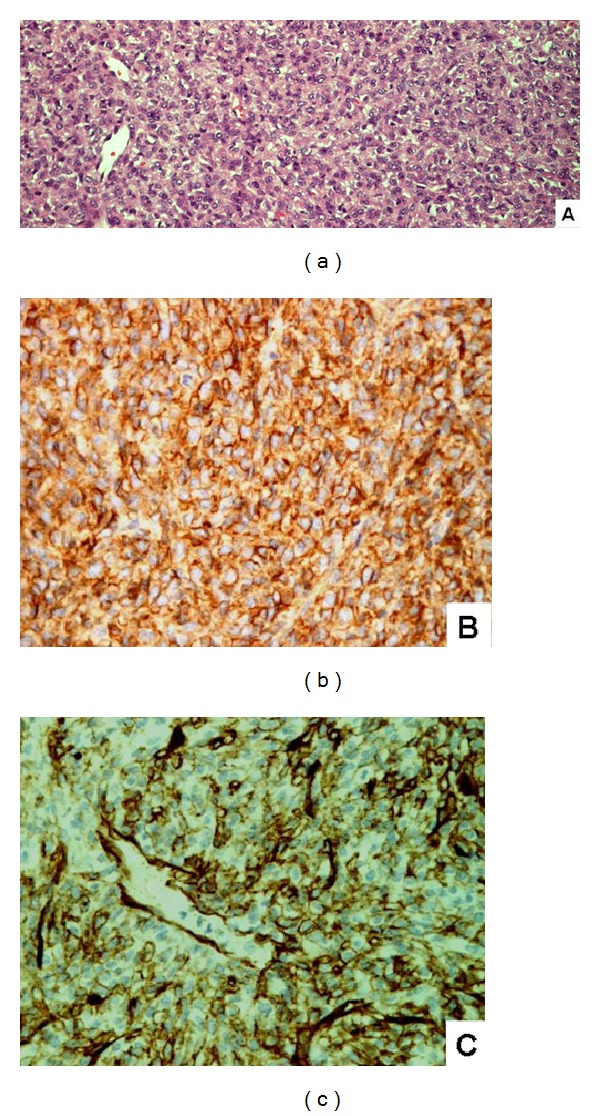
Histology and immunohistochemistry of meningeal hemangiopericytoma. (a) H&E 20x A hypercellular tumor with staghorn blood vessels. (b) Immunostaining of CD99 is diffuse and strong positive. (c) Immunostaining of CD 34 reveals variable positive.

Microscopically, the recurrent meningeal lesion showed a hypercellular tumor intermingled with focal necrosis. The tumor consists of nonanaplastic small round or oval cells with pale nuclei, distinct nucleoli, and inconspicuous cytoplasm. Mitotic count ranges from 5 to 8 per 10 high power fields. Staghorn or ectatic blood vessels are seen in some areas (Figure 2(a)).

Immunohistochemically the neoplastic cells characteristically express diffusely CD99 (Figure 2(b)) and bcl2 and variable CD34 (Figure 2(c)), whereas no immunoreactivity was seen for EMA or progestogen receptor. The proliferative index indicated by MIB-1 (Ki67) is less than 1%.

On the basis of the morphological and immunohistochemical staining features, a final diagnosis of recurrent meningeal hemangiopericytoma was made.

 After 1 year of the final operation, the patient presented with abnormal behavior with hypoglycemia symptoms such as nervousness, sweating, trembling, weakness, palpitation, and often had trouble in speaking. Hepatic and renal function test was unremarkable. Serum test for sugar glucose, insulin, growth hormone, and insulin-like growth factor-1 was low except insulin-like growth factor-2 in “normal range” (Table 1). No oral GTT test was done. CT demonstrated residual tumor at right posterior fossa while metastatic nodular enhancing masses presented at bilateral kidney, right iliopsoas muscle, right iliac body, thoracic vertebra 10 body and segment IV of the liver. Clinically diagnosed as recurrent meningeal hemangiopericytoma with multiple metastasis and hypoglycemia of non-islet cell tumor, the patient was treated with dexamethasone for hypoglycemia, adriamycin for the tumor. He subsequently felt well, and his sugar glucose returned to normal. The patient is still alive. Last follow-up is in May with MRI in March 2012. The MRI reported that the number and size of extra-axial enhancing mass lesions are increased, compared with previous MRI, suggestive of disease progression.

## 3. Discussion

Meningeal hemangiopericytoma is a rare neoplasm, but it possesses rather high recurrent rate in 60.6% [3] and 80% [4], respectively. It is also a major brain tumor that possesses extracranial metastasis. The metastatic rate of this tumor is 23.4% [3] and worse to 68% at 15 years [5]. Most of primary brain tumor, such as glioma and meningioma, rarely exhibits extracranial metastasis though in malignant form. However, the majority of meningeal hemangiopericytoma can eventually metastasize elsewhere in body. 

The aberrant presence of hypoglycemia produced by the tumor strongly suggests extracranial metastasis of meningeal hemangiopericytoma. Daughady et al. [6] reported mesenchymal tumor could secret insulin-like growth factor-II (IGF-II) that can induce hypoglycemia. However, such situation in brain mesenchymal tumor is rare. To our knowledge, only 5 cases of hypoglycemia were until now reported in meningeal hemangiopericytoma [7–11]. Multiple factors can induce hypoglycemia of nonislet cell tumor issue. Good balance of GH, insulin, IGF-I, and IGF-II is essential to stable normal biochemistry and physiologic function [12]. In our case, however, this balance was disturbed by (1) excessive glucose consumption by the voluminous tumoral mass, especially by metastatic one [13]. (2) Tumor produces pro-IGF-II peptide or big IGF-II [7, 14] which is different from normal IGF-II with high molecular weight. (3) Tumor producing complex IGF-II bound to IGF binding protein 2 (IGFBP 2) about 40 kilodaltons in circulation, instead of normal complex IGF-II bound to IGF binding protein 3 (IGFBP3) and acid-labile glycoprotein about 150 to 200 kilodaltons. This low weight complex can easily permeate capillary membranes and cause hypoglycemia by a direct interaction with IGF and insulin receptors [8, 10, 14, 15]. Hypoglycemia induced by elevated tumorous IGF-II can produce a negative feedback and cause GH, insulin, and IGF-I downregulation. In our case, low levels of GH, insulin and IGF-I were detected, though IGF-II was in normal range. In this instance, the normal range of IGF-II should be considered as “inappropriate normal” because of others in low levels. Subsequently, blood glycogen, insulin, and IGF-I are almost recovered after target treatment to the primary and metastatic lesion of our case. This result demonstrates when hypoglycemia happens in a meningeal hemangiopericytoma, metastasis of the tumor should be considered. 

## Figures and Tables

**Table 1:** Serum levels of the patient when presented with hypoglycemia.

	Patient	Normal range
Insulin (mIU/L)	<0.2	2.6–24.9
C-peptide (nmol/L)	0.03	0.27–1.27
Sugar Glucose (mmol/L)	2.1	3.9–6.2
GH (mIU/L)	<0.3	N/A
IGF-I (nmol/L)	<3.5	13.2–34.9
IGF-II (ng/mL)	602	288–736

GH: growth hormone, IGF-I: insulin-like growth factor-I, IGF-II: insulin-like growth factor-II. N/A: not available.
